# Injectable corneal endothelial cell therapy: recent progress, translational barriers, and future directions

**DOI:** 10.3389/fopht.2026.1799892

**Published:** 2026-05-08

**Authors:** Ghadeer Mohammed, Muhammad Abbas Tayyab, Jaffer Hussain, Renad AlDhib, Mifrah Rahat Khan Sherwani, Johar Abbas, Zahra Arsalan, Carolina Mercado

**Affiliations:** 1 University of Kufa College of Medicine, Najaf, Iraq; 2 King Edward Medical University, Lahore, Pakistan; 3 MAHSA University, Selangor, Malaysia; 4 Duke University Hospital, Durham, NC, United States; 5 Al-Shifa Trust Eye Hospital, Rawalpindi, Pakistan; 6 Bascom Palmer Eye Institute, Naples, FL, United States

**Keywords:** cell therapy, corneal endothelial dysfunction, fuchs endothelial corneal dystrophy (FECD), human corneal endothelial cells (HCECs), injectable endothelial cell transplantation, pseudophakic bullous keratopathy (PBK)

## Abstract

**Introduction:**

Corneal endothelial dysfunction, most commonly caused by Fuchs endothelial corneal dystrophy (FECD) or pseudophakic bullous keratopathy (PBK), leads to stromal edema and corneal decompensation when endothelial cell density (ECD) falls below 500–700cells/mm². Standard treatment via corneal transplantation, including Descemet stripping automated endothelial keratoplasty (DSAEK) and Descemet membrane endothelial keratoplasty (DMEK), is limited by the need to standardize corneal endothelial cell culture, donor shortages, immune rejection, and technical complications.

This review aims to summarize recent advances in injectable corneal endothelial cell (CEC) therapy, critically appraise translational challenges, and discuss future directions for establishing this regenerative approach as a global treatment.

**Design:**

Narrative review of pre-clinical and clinical studies examining CEC injection therapy.

**Methods:**

Relevant literature was analyzed to evaluate innovations in Human corneal endothelial cells (HCEC) preparation, culture techniques, delivery methods, and strategies to enhance cell adhesion and survival. Studies reporting preclinical models and human clinical trials were included to assess safety, efficacy, and translational feasibility.

**Results:**

Corneal endothelial cells (CEC) injections have achieved significant advancement since their development. Simple cultured CEC injections showed poor efficacy due to limited cell adhesion and survival, but the introduction of ROCK inhibitors along with cultured CECs demonstrated improvement in corneal transparency up to 5 years. Advancements in cell delivery techniques like hydrogel, carrier-assisted injections, magnetically guided injections, as well as alternative cell sources have shown promising results in pre-clinical studies, but human studies are still ongoing. Despite advancements, persisting challenges include phenotypic stability, and longterm safety and efficacy.

**Discussion:**

Injectable CEC therapy is a promising minimally invasive alternative to corneal transplantation. While early clinical outcomes are encouraging, further work is required to optimize cell preparation, delivery, and long-term safety, and to establish the therapy as a scalable, globally applicable treatment for endothelial failure.

## 1 Introduction

The human corneal endothelial cells (HCECs), a single layer of hexagonal cells, are crucial for maintaining corneal transparency. It regulates stromal hydration through active ionic pumps and barrier mechanisms (1). Dysfunction or loss of HCEC disrupts this balance, leading to stromal edema, progressive visual impairment, and ultimately, corneal decompensation (2, 3). Endothelial failure, caused by Fuchs endothelial corneal dystrophy (FECD) and pseudophakic bullous keratopathy (PBK) (4), occurs when the endothelial cell density (ECD) drops below the critical threshold of approximately 500–700 cells/mm^2^ (2, 5).

Endothelial keratoplasty, particularly Descemet’s Membrane Endothelial Keratoplasty (DMEK) and Descemet’s Stripping Automated Endothelial Keratoplasty (DSAEK), currently represents the standard of care for endothelial failure. It has improved clinical outcomes, but its scalability and efficacy are constrained by several critical limitations (5–7). To overcome the challenges inherent in traditional transplantation, injectable corneal endothelial cell (CEC) therapy has emerged as a promising, minimally invasive, and regenerative alternative. This approach involves injecting cultured CECs often supplemented with Rho-associated kinase (ROCK) inhibitors, which promote endothelial cell adhesion, survival, and functional recovery by modulating cytoskeletal signaling (8, 9), as well as addressing the need for scalable cell sources, such as induced pluripotent stem cell (iPSC)-derived CECs (10, 11).

**Figure 1:**
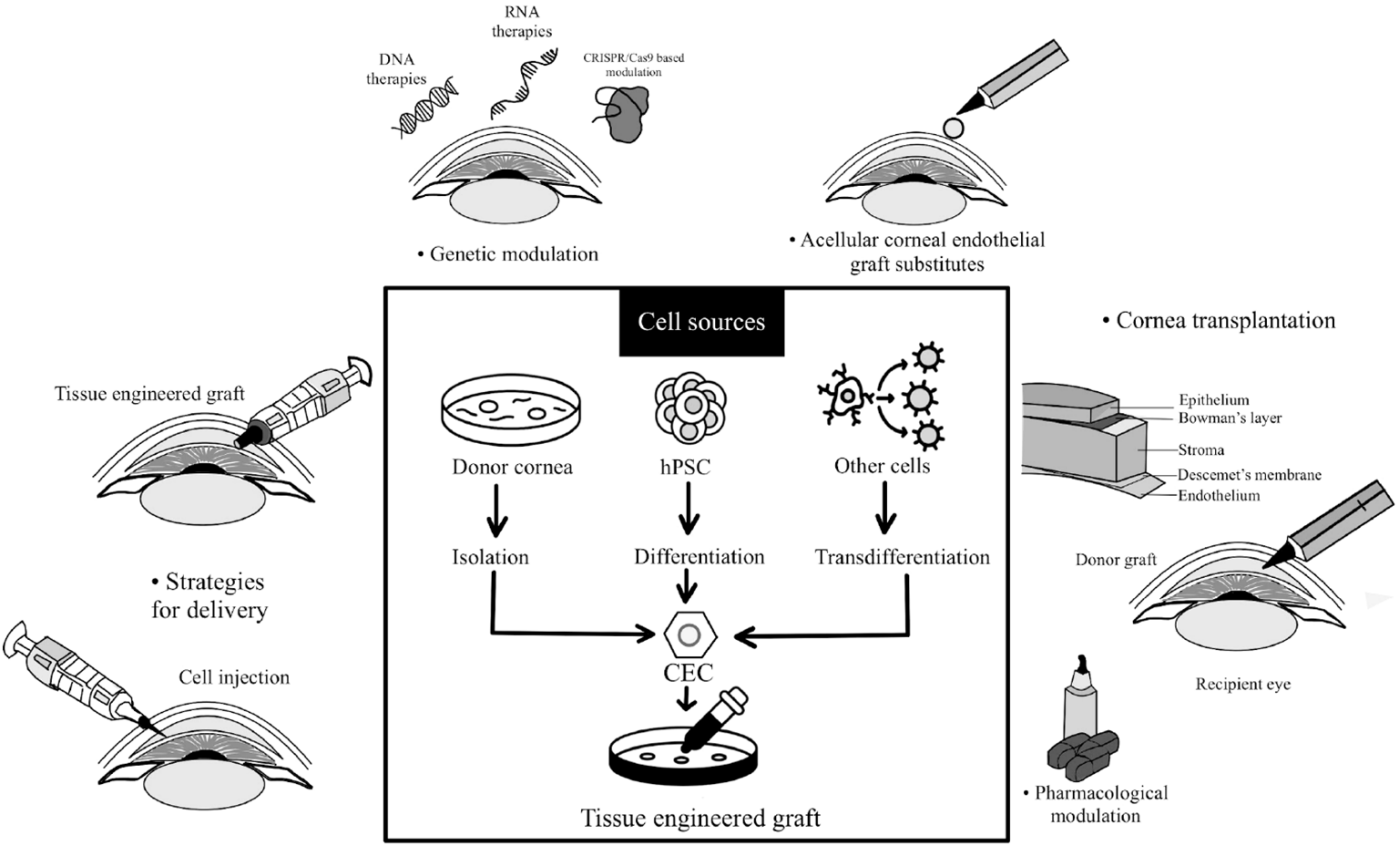
Corneal endothelial regeneration strategies include transplantation, cell therapies, acellular grafts, and modulation of the endothelium through pharmacological or genetic means, offering potential solutions for restoring corneal health and vision.

This narrative review was undertaken to synthesize and critically appraise current and prospective evidence on corneal endothelial cell (CEC) injection therapies, including progress in cell preparation, expansion methods, and delivery strategies. It also evaluates key translational challenges such as phenotypic stability and regulatory standardization and highlights future directions needed to establish CEC injection as a scalable global therapy (**Figure 1**) While previous reviews have discussed corneal endothelial cell biology and regenerative approaches, many mainly focus on preclinical work or specific areas like biomaterials and cell culture (3, 8). As a result, they often miss the full clinical picture.

This review takes a broader approach by combining recent clinical trial data with advances in cell delivery, biomaterials, and stem cell–derived endothelial cells. It also highlights practical issues such as safety, regulatory challenges, outcome variability, and scalability.

Overall, it aims to link research with real clinical use and provide a clearer view of the current state of CEC injection therapy.

## 2 Methods

### 2.1 Literature search strategy

This study was conducted as a narrative review aimed at providing a comprehensive overview of the current evidence on corneal endothelial cell (CEC) injection therapy and related regenerative approaches. An extensive literature review was conducted to search for peer-reviewed journals, clinical trials, reviews, and guidelines regarding corneal endothelial cell injection therapy. Study selection was performed through title and abstract screening followed by full-text review of potentially eligible articles. Studies were included based on relevance to corneal endothelial cell injection therapy, including cell sources, delivery strategies, and clinical or translational outcomes. Approximately 45 studies were included in this review which were further grouped into predefined categories:

1. 14 Preclinical studies (*In-vitro* and animal models).
2. 6 Human interventional studies (early-phase clinical trials).
3. 1 Observational/registry studies.
4. 24 Reviews and guidelines (used for contextual background).

The databases PubMed, Scopus, and Google Scholar were searched, including relevant articles published from January 2000 up to September 2025, to capture both the seminal and recent evidence.

The following terms and Medical Subject Headings (MeSH) were utilized in combinations:

“corneal endothelial cells”, “corneal endothelial cell injection”, “cell-based therapy”,

“regenerative ophthalmology”, “ROCK inhibitor”, “iPSC-derived endothelial cells”,

“intracameral cell injection”, “Descemet’s membrane”, “corneal transplantation”, “DMEK”, “DSAEK”, and “Fuchs endothelial corneal dystrophy”. The reference lists of the included articles were also manually screened to detect additional relevant studies.

### 2.2 Inclusion and exclusion criteria

Quality publications were original research articles, systematic reviews, meta-analyses, narrative reviews, and large clinical trials published in the English language. Publications were included if they mentioned cell sources, expansion strategies, delivery methods, clinical uses, or outcomes of corneal endothelial cell therapy.

Exclusion criteria involved conference abstracts with minimal data, individual case reports, animal models without translational relevance, non-peer-reviewed documents, and publications that were not in the English language.

### 2.3 Data extraction and synthesis

Information was manually extracted from the included studies, including study design, sample, type of corneal disease, source of cells, delivery method, adjunctive treatments, and clinical or histological outcomes. Results were synthesized narratively and structured into the most important thematic sections including the history of development, biological and technological progress, delivery approaches, clinical results, and future directions.

Critical evaluation centered on finding therapeutic advancement, shortcomings of existing methods, translational hurdles, and promising innovations like bioengineered scaffolds, ROCK inhibitors, and endothelial cells derived from stem cells. The integration was to enable a balanced and inclusive perspective of the existing scenario and upcoming possibilities in corneal endothelial cell regeneration.

## 3 History and developmental techniques

Over the past few decades, there has been a significant transformation in the treatment of corneal endothelial dysfunction evolved from purely symptomatic medical therapies, including topical hyperosmotic, intraocular pressure lowering agents, steroids, and antibiotics, which alleviate discomfort but do not restore endothelial function (12, 13). Penetrating keratoplasty provided the first definitive surgical solution but was limited by graft rejection, prolonged visual rehabilitation, and structural compromise (14, 15). These limitations drove the development of selective endothelial replacement techniques, beginning with Descemet stripping endothelial keratoplasty (DSEK) and DSAEK, which improved visual recovery and reduced immunologic risk. Subsequent refinement led to DMEK, now regarded as the surgical gold standard due to superior visual outcomes and lower rejection rates (14, 15). However, persistent dependence on donor tissue, technical complexity, and limited global accessibility have catalyzed the emergence of minimally invasive regenerative approaches, most notably corneal endothelial cell injection therapy, as a potential donor-independent alternative (14, 15).

Studies in the 1990s and early 2000s confirmed the proliferative ability of CECs *in vitro* maintaining the structural and functional characteristics of cells, but culture media properties, delivery techniques, survival, and adhesion of injected endothelial cells in the host remained challenging (16). Preclinical studies by Okumura et al. demonstrated that intracameral injection of cultured corneal endothelial cells in combination with the ROCK inhibitor Y-27632 resulted in regeneration of a hexagonal endothelial monolayer expressing key barrier- and pump-related proteins in a primate model (5). This addition significantly enhanced endothelial cell adhesion and survival on the host Descemet membrane, by promoting proliferation and adhesion directly into the anterior chamber, where they repopulate the posterior corneal surface and restore pump function (2, 8). Initial studies in animals and recent landmark human trials have demonstrated that this regenerative strategy can safely restore corneal clarity and visual acuity (9, 10). These studies reported regeneration without adverse effects such as rejection, secondary glaucoma, or ectopic cell transplantation, addressing a major limitation of early cell-injection approaches (17, 18). However, major translational challenges remain, specifically in maintaining the phenotype of cultured cells, ensuring the structural integrity of the recipient’s Descemet’s membrane. Early clinical evidence from Kinoshita et al. (19) demonstrated that HCEC injection therapy can safely restore corneal transparency and endothelial function in patients with bullous keratopathy. In the first-in-human Phase I clinical study, 11 eyes received an intracameral injection of 1 × 10^6^ cultured CECs supplemented with the ROCK inhibitor Y-27632, followed by 3 hours of prone positioning. By 24 weeks, all treated eyes achieved restoration of corneal transparency, with central endothelial cell densities exceeding 500 cells/mm² (range, 947–2833 cells/mm²), and no treatment-related serious adverse events were reported (19). These findings provided the first clinical evidence supporting the safety of this approach.

Furthermore, long-term follow-up data demonstrated that corneal clarity, visual acuity, and endothelial function remained stable for up to five years. At 5 years post-treatment, normal corneal endothelial function was restored in 10 of the 11 treated eyes, with a mean ± SD ECD of 1257 ± 467 cells/mm² (range, 601–2067 cells/mm²), significant improvement in best corrected visual acuity (BCVA) in 10 eyes, and no major adverse reactions related to CEC injection therapy (5). This indicates the efficacy and long-term safety of this therapy. In parallel with CEC-based strategies, other cell sources have been explored for corneal regeneration. Mesenchymal stem cells (MSCs) are multipotent adult stem cells obtainable from sources such as bone marrow, adipose tissue, and umbilical cord. MSCs have been shown to differentiate toward corneal cell lineages and contribute to tissue repair through paracrine and immunomodulatory mechanisms (20, 21). De Miguel et al. emphasize that MSCs are increasingly used for corneal layer-specific regenerative approaches due to their immunomodulatory properties and clinical translational potential (20, 21). Following this breakthrough, subsequent research has focused on optimizing delivery precision and maximizing early cell retention.

### 3.1 Delivery techniques

Effective delivery of cultured corneal endothelial cells (CECs) to the posterior cornea remains a key factor in therapeutic success. Injection-based approaches, particularly in combination with ROCK inhibitors, have demonstrated improved cell adhesion and survival in both preclinical and clinical studies (5), while other strategies aim to optimize structural support and integration (3). These developments reflect ongoing efforts to overcome the limitations of earlier techniques, including poor cell retention, limited adhesion, and challenges in maintaining functional endothelial monolayers following transplantation (3).

Initial strategies focused on sheet-based or scaffold-assisted transplantation, in which cells establish intercellular junctions and organize into continuous sheets that closely resemble the native endothelium (20). Despite this favorable organization, engineered CEC sheets are inherently fragile and cannot be manipulated safely during surgery without additional mechanical support (20). An effective carrier for CEC therapy should replicate the essential structural and functional properties of native Descemet’s membrane, including optical transparency, permeability to nutrients and small molecules, appropriate mechanical strength, flexibility to conform to the posterior corneal curvature, and biocompatibility to support endothelial cell adhesion and survival (22). Equally important is the ability of the CEC carrier construct to achieve stable and durable adhesion to the posterior corneal surface, because long-term graft success depends on proper integration with the host tissue. Clinical experience from DMEK shows the importance of biomechanical compatibility between the transplanted tissue and the recipient cornea, as anatomical replacement of Descemet’s membrane and endothelium enables restoration of near-native corneal structure and function (23). However, the strict requirements for mimicking Descemet’s membrane and the difficulty of handling fragile endothelial cell sheets, have limited the widespread clinical adoption of sheet-based strategies and shifted the focus toward less invasive delivery approaches (23).

Against this background, CEC injection therapy emerged as a simpler and minimally invasive alternative in which dissociated cultured CECs are injected into the anterior chamber, where gravity-assisted positioning, together with ROCK inhibition, enhances cell adhesion, survival, and spreading on Descemet’s membrane (5, 19). By avoiding complex graft manipulation, this strategy substantially reduces surgical complexity while preserving functional endothelial regeneration, as demonstrated in early clinical studies by Kinoshita and colleagues (19). In preclinical models, magnetic nanoparticle-guided CEC injection enabled targeted deposition of transplanted cells onto Descemet’s membrane and achieved approximately a 2.4-fold improvement in attachment efficiency compared with gravity-assisted methods (≈86% vs 36%) (24, 25). More recently, hybrid delivery strategies have been introduced to enhance early functional recovery while retaining the minimally invasive nature of injection therapy. Notably, Ishino and colleagues (26) described a mini-sheet injection technique in which small, ordered CEC monolayers are co-delivered with ROCK inhibitors. This approach improves cell adhesion and accelerates re-endothelialization, effectively bridging the gap between dissociated cell injection and conventional sheet-based transplantation.

Taken together, these developments have reshaped the field of corneal endothelial regeneration, shifting the therapeutic paradigm away from complex graft-replacement surgery toward a precise, injectable, and potentially scalable cell-based treatment. **Table 1** summarises the progress in cell-carrier design, highlighting the advantages and limitations associated with various substrate materials.

**Table 1:** Progress in cell-carrier design for corneal endothelial cell therapy.

Carrier type	Key advantages	Key limitations	Representative references
Collagen/Denuded Descemet’s membrane	1. Native-likemicroenvironment for CEC attachment and monolayer formation. 2. Preserves hexagonal morphology and pump function in ex vivo/preclinical work. 3. Good biocompatibility for engraftment.	1. Limitedmechanical strength; risk of variable thickness. 2. Enzymaticdegradation/handling difficulty. 3. Surgical manipulation can be delicate.	Gutermuth et al., Cornea 2019 (31). (Descemet’s-like microtopography & differentiation).; Bosch et al., Regenerative Biomaterials 2022 (32).
Gelatin-based hydrogels	1. Highbiocompatibility; supports cell adhesion/survival. 2. Tunablestiffness/porosity/degra dation. 3. Surface chargemodification can enhance integrinmediated attachment.	1. Crosslinkingvariability affects mechanical properties. 2. Long-term invivo degradation/transpar ency not fully characterized. 3. Requires balanceto avoid opacity.	Lai et al., Biomacromolecules 2006 (effect of charge & molecular weight on gelatin carriers).
Thermosensitive hydrogels	1. Sol→gel transition simplifies surgical delivery. 2. Maintains cornealtransparency in animal models. 3. Improves survival ofiPSC-derived CECs in preclinical studies.	1. Limited longterm human safety/efficacy data. 2. Possible batch variability. 3. Requires precise temperature control for gelation.	Chi et al., Acta Biomaterialia 2025 (29) (thermosensitive hydrogel + iPSCderived CECs, rabbit model).
Biomimetic substrates	1. Guides stem celldifferentiation to an endothelial-like phenotype. 2. Mimics Descemet’smembrane topography. 3. Provides stable, biologically functional support.	1. Complex manufacturing. 2. Scalability challenges. 3. Long-term *in vivo* performance not fully tested.	Gutermuth et al., Cornea 2019 (31); Bosch et al., Regenerative Biomaterials 2022.
Nanostructured carriers/ nanoparticle-assisted delivery	1. Control over cellorientation/positioning. 2. Improvedretention/adhesion of transplanted cells. 3. Enables approachessuch as magnetic guidance.	1. Safety of nanoparticles in the human eye requires further validation. 2. Complex synthesis and cost. 3. Need optimization for biodegradability and biocompatibility.	Moysidis et al., Nanomedicine 2015 (24); Xia et al., IOVS 2019 (25) (magnetic guidance studies).

### 3.2 Cell carriers

Optimizing cell-carrier design has emerged as a central consideration in improving engraftment efficiency and long-term therapeutic performance in corneal endothelial regeneration. Early carrier systems, including collagen membranes and denuded Descemet’s layers, provided important initial insights but were limited by variability in material properties, handling challenges, and inconsistent biological outcomes (23). These limitations prompted a shift toward bioengineered materials designed to more closely reproduce the native basement membrane microenvironment of the corneal endothelium (27).

Among the most extensively studied platforms are gelatin-based hydrogels, derived from partially degraded collagen and engineered to allow precise control over molecular weight, charge distribution, stiffness, porosity, and degradation kinetics. Their positively charged surfaces promote attachment of CEC via integrin-mediated interactions and help to maintain the ionic balance required for proper Na^+^/K^+^-ATPase activity and fluid regulation (28). In preclinical models, both gelatin methacrylate (GelMA) and oxidized gelatin crosslinked with genipin achieved over 90% cell viability, strong ZO-1 and Na^+^/K^+^-ATPase expression, and sustained corneal transparency and hydration balance in rabbit and porcine studies (29). Building on the success of these materials, thermosensitive hydrogels have been introduced to further simplify surgical handling and enhance post-transplantation cell survival (3, 29). These systems remain injectable at lower temperatures and undergo *in situ* gelation under physiological conditions, enabling precise placement of transplanted cells while minimizing mechanical stress during delivery (29, 30). In animal models, thermosensitive hydrogels loaded with iPSC-derived endothelial cells have demonstrated improved graft integration and corneal clarity, supporting their potential role in minimally invasive delivery strategies (29).

In parallel, increasing emphasis has been placed on biomimetic carrier designs that replicate the ultrastructural features of native Descemet’s membrane (31, 32). Substrates patterned with Descemet’s membrane-like microtopography have been shown to direct stem-cell differentiation toward an endothelial-like phenotype while maintaining sufficient mechanical stability for transplantation (30, 33, 34). By integrating topographical cues with appropriate material properties, these carriers provide both structural support and biological signaling necessary for functional endothelial maturation (31, 32).

More recently, nanostructured carrier systems have expanded the functional capabilities of cell delivery platforms. Magnetically responsive and silica-based scaffolds offer additional control over cell orientation, spatial positioning, and early retention at the target site, while maintaining favorable safety profiles in preclinical studies (25, 32). When combined with external guidance strategies, such as gravity-assisted positioning, air tamponade, or magnetic targeting, these systems enable more precise localization of transplanted cells to the posterior cornea and further improve engraftment efficiency (35, 36).

Taken together, these developments reflect a growing convergence of materials science and regenerative ophthalmology. Modern cell carriers are increasingly designed not as passive supports, but as active modulators of cell adhesion, survival, differentiation, and integration, thereby playing a pivotal role in advancing reliable and scalable cell-based therapies for corneal endothelial disease.

### 3.3 Future directions

Despite rapid progress, several barriers still prevent widespread adoption of CEC injection therapy like unified cell-source development, carrier design, and regulatory standardization (27, 33). Consistency in manufacturing, long-term genomic safety of iPSC-derived cells, and cross-regional quality-control guidelines remain major challenges (11). Randomized controlled trials comparing CEC injection therapy with established endothelial keratoplasty techniques are critically needed to define comparative efficacy and cost-effectiveness.

Continued collaboration among cell biologists, materials engineers, and clinicians will be vital to fully realize donor-independent regenerative treatments. As refinement of delivery systems and carriers continues, the goal is to achieve a durable, reproducible, and globally accessible therapy capable of restoring vision with the same reliability once expected only from keratoplasty. The key clinical milestone and landmark studies in the evaluation of this therapy is summarised in **Table 2**.

**Table 2:** Key milestones in CEC injection therapy.

Study (phase)	Key findings	Representative citation
Okumura et al. (preclinical studies on ROCK inhibitors and CEC biology)	ROCK inhibitors (Y27632) enhanced CEC adhesion, proliferation, and survival *in vitro*; promoted wound healing in animal models.	Okumura N., Koizumi N., Ueno M., et al. IOVS 2009 (ROCK inhibitor enhances primate CEC survival). Also Okumura et al., Br J Ophthalmol 2011 (17) (ROCK inhibitor eye drops accelerate wound healing).
Kinoshita et al. (first human intracameral cell injection trial)	Intracameral delivery of cultured CECs with ROCK inhibitor improved corneal clarity, ECD, and visual acuity in patients with bullous keratopathy (first-inhuman NEJM trial).	Kinoshita S., Koizumi N., Ueno M., Okumura N., Imai K., Tanaka H., et al., NEJM 2018 (Injection of cultured cells with ROCK inhibitor for bullous keratopathy).
Numa et al. (long-term follow-up)	Five-year follow-up of initial patients showed sustained corneal clarity and stable ECD without immune rejection in many cases.	Numa K., Imai K., Ueno M., et al., Ophthalmology/Am J Ophthalmol 2021 Five-year follow-up.
Kobayashi et al. (Vyznova early clinical outcomes/multicenter trials)	Early multicenter results show consistent clinical outcomes; promising safety and reproducibility for cultured CEC injection (Vyznova).	Kobayashi A., Mori N., Yokogawa H., Sugita S., et al., Cornea (Vyznova early clinical outcomes) 2025.
Hirayama et al. (iPSC/allogeneic iPSCderived CEC substitute firstin-human)	Transplantation of allogeneic iPSCderived CEC substitute restored corneal transparency in early follow-up without tumor formation in the reported 1-year data.	Hirayama M., Hatou S., Nomura M., et al., Cell Reports Medicine 2025 (11) first-in-human iPSC-derived CEC substitute study.

## 4 Discussion

CEC injection therapy represents a significant advancement in regenerative ophthalmology, providing a minimally invasive alternative to conventional keratoplasty. Originally developed as an experimental approach, this therapy has progressed to clinical validation, with sustained outcomes reported in human trials, most notably in Japan. By restoring corneal clarity without full graft replacement, CEC injection therapy expands treatment options for corneal endothelial dysfunction and has the potential to reduce reliance on donor tissue and technically demanding surgery (5, 22, 37). However, formal analyses addressing cost-effectiveness and healthcare system integration remain limited (19, 22, 38–40).

Endothelial keratoplasty procedures, particularly Descemet membrane endothelial keratoplasty (DMEK) and Descemet stripping automated endothelial keratoplasty (DSAEK), remain the current standard of care for corneal endothelial dysfunction due to their well-established efficacy, predictable visual outcomes, and long-term graft survival (7, 34). These techniques involve anatomical replacement of the diseased endothelium and Descemet’s membrane, allowing rapid restoration of corneal clarity and function. While CEC injection therapy represents a fundamentally different regenerative approach, relying on *in vivo* adhesion, proliferation, and functional integration of injected cells (5, 22). This strategy offers potential advantages, including reduced surgical complexity and the possibility of scalable, donor-independent treatment (22, 39). However, unlike DMEK, which provides immediate structural replacement, CEC injection outcomes depend on successful cell engraftment and may demonstrate variability in endothelial cell density and clinical response (5, 41). Importantly, no studies to date have directly compared CEC injection therapy with DMEK or DSAEK in randomized settings, limiting definitive conclusions regarding relative efficacy and long-term durability (27, 33).

Nevertheless, recent studies have expanded both the cellular sources and delivery strategies used in endothelial regeneration. A major milestone was achieved by Hirayama et al. (11), who reported the first human transplantation of allogeneic pluripotent stem cell–derived CECs in a patient with bullous keratopathy, demonstrating improved corneal transparency without immune rejection or tumor formation at one year of follow-up. This study highlighted the feasibility of donor-independent cell sources for endothelial repair. Supporting *in vitro* and animal studies have shown that CECs derived from hESC and MSC exhibit differentiation capacity and partial functional comparability, further broadening the therapeutic landscape (4, 42).

Parallel advances in delivery technologies have aimed to improve cell targeting, adhesion, and survival. While engineered CEC sheets can replicate native endothelial architecture, their fragility and surgical complexity limit clinical practicality (24). Consequently, preclinical research has focused on injection-based and carrier-assisted strategies. Magnetic nanoparticle guided delivery enables targeted deposition of CECs without altering cellular morphology or function (24), thermosensitive hydrogels provide injectable, biocompatible support that enhances cell retention and survival (29), and bioengineered scaffolds including extracellular matrix coatings and functionalized synthetic polymers create optimized microenvironments that support CEC expansion and phenotypic maintenance (3). More recently, microcarrier-based systems and AI-guided delivery methods have further improved precision in cell targeting and early engraftment (43).

Despite these advances, important biological and translational challenges persist. Cultured CECs exhibit limited proliferative capacity and phenotypic instability, even with the use of ROCK inhibitors, which primarily enhance survival and adhesion rather than long-term self-renewal (33, 46). In addition, variability in differentiation among iPSC- and hESC-derived CECs raises concerns regarding tumorigenic risk, sensitivity to culture conditions, and the lack of standardized manufacturing protocols.

Clinically, the available evidence remains encouraging but limited. Most studies have been single-center and small-scale, predominantly conducted in Japan, restricting generalizability. Nevertheless, reported outcomes consistently demonstrate improvements in corneal clarity, BCVA and ECD, with low rates of serious adverse events. Long-term follow-up beyond five years is available from only a single study by Numa et al. (41). Excellent corneal restoration with good BCVA was maintained in 10 of the 11 treated eyes in the study. One eye with pseudo exfoliation-related endothelial failure showed initial improvement, with an ECD of 871 cells/mm² at 2 years, followed by the development of mild corneal stromal edema at 3 years that did not progress by year 5. Two successful surgical outcomes demonstrated sustained corneal transparency and thickness in one patient with FECD and another with argon laser iridotomyrelated endothelial failure. No local or systemic adverse events, such as immunologic endothelial rejection, uveitis, or infection, were reported. A transient steroid-induced increase in intraocular pressure occurred in one patient and was successfully managed. Variability in postoperative endothelial cell density and occasional decline in long-term cell function were also observed (41). Available evidence indicates that while corneal endothelial cell injection therapy demonstrates encouraging safety and efficacy, available data remain limited and derived primarily from small, single-center cohorts and has been reported to be associated with potential adverse events, including cell loss, phenotypic instability, and unpredictable long-term cellular behavior, underscoring the need for cautious clinical translation (18).

Long-term follow-up data further revealed that not all treated eyes maintained durable endothelial function, with at least one case showing recurrent stromal edema several years after an initial improvement, indicating the possibility of late or partial therapeutic failure (41). Likewise, although corneal clarity was restored in treated eyes, a wide variability in postoperative ECD was observed, suggesting heterogeneous clinical responses rather than uniform success (5). Taken together, these findings support the view that early clinical success should be interpreted cautiously, as variability in cell phenotype, manufacturing consistency, and clinical response introduces a tangible risk of treatment failure during broader clinical adoption (33).

In this context, it is particularly important to note that no studies to date have directly compared CEC injection therapy with established keratoplasty techniques such as DMEK or DSAEK, limiting definitive assessment of relative efficacy and durability (27, 33).

Future research should therefore prioritize standardization, scalability, and comparative evaluation. Integration of advanced quality-control tools, including transcriptomic profiling and live-cell imaging, may improve potency assessment and manufacturing consistency (44). In this context, the AI-based U-Net model introduced by Okumura et al. for monitoring cultured CEC morphology represents an important step toward automated quality assurance (43). Ultimately, large, multicenter trials with direct comparisons to conventional keratoplasty will be essential to define the long-term safety, clinical positioning, and global applicability of CEC injection therapy.

## 5 Conclusion

CEC injection therapy has rapidly evolved from laboratory innovation to a viable clinical alternative to endothelial keratoplasty. The introduction of ROCK inhibitors enabled reliable adhesion and functional recovery, leading to sustained corneal clarity and stable ECD for up to five years. Although challenges persist particularly in standardizing cell preparation, ensuring genomic safety of stem-cell–derived CECs, and establishing global manufacturing protocols, ongoing advances in delivery systems and donor-independent cell sources continue to expand the therapeutic potential of this technology. CEC injection therapy represents a transformative step toward minimally invasive, scalable, and donor-independent treatment for corneal endothelial diseases.
